# Efficacy and Safety of Insulin Degludec/Insulin Aspart (IDegAsp) in Type 2 Diabetes: Systematic Review and Meta-Analysis

**DOI:** 10.7759/cureus.25612

**Published:** 2022-06-02

**Authors:** Brenda C Edina, Jeremy R Tandaju, Lowilius Wiyono

**Affiliations:** 1 Faculty of Medicine, Universitas Indonesia, Jakarta, IDN

**Keywords:** meta-analysis, systematic review, insulin, diabetes mellitus type 2, idegasp

## Abstract

Type 2 diabetes mellitus is a prevalent metabolic disease requiring tight glycemic control of basal and postprandial glucose levels. Treatment intensification using separate basal and bolus injections increased the number of injections and reduced cost-effectivity, leading to decreased compliance and failure of glycemic control. Insulin Degludec/Insulin Aspart (IDegAsp), a novel premix of basal and bolus insulin, is one of the potential treatments for reducing the number of injections. However, its efficacy and safety have not been reviewed clearly. Therefore, this systematic review aims to compare the efficacy and safety of IDegAsp with standard basal and basal plus bolus insulin regimens.

A systematic review of four databases (Pubmed, Scopus, Science Direct, and Proquest) was conducted using the Preferred Reporting Items for Systematic Reviews and Meta-Analyses (PRISMA) guideline. Search results were screened by eligibility criteria and critically appraised by the Oxford Centre for Evidence-Based Medicine (CEBM) tool and the Cochrane risk-of-bias* a*ssessment tool. Meta-Analysis was done using Review Manager to obtain cumulative outcomes from hemoglobin A1C (HbA1C) changes, hypoglycemia incidents, and weight gain from all studies.

Out of 132 search results, 10 studies were reviewed. IDegAsp once-daily administration was proven beneficial in reducing HbA1c levels and nocturnal hypoglycemia incidences, while IDegAsp twice-daily administration was proven beneficial in lowering hypoglycemia incidence and nocturnal hypoglycemia incidence. IDegAsp yielded better glycemic index results and lowered hypoglycemic incidents in the meta-analysis.

Thus, it is concluded that IDegAsp once daily with stepwise titration on the largest meal of the day achieved most benefits with minimal risks.

## Introduction and background

Diabetes mellitus is a metabolic disease suffered by 422 million people globally (2014) and a direct cause of 1.6 million mortalities (2016) due to its complications [1]. Type 2 diabetes mellitus (T2DM), a subtype of diabetes mellitus more prevalent in adults, is characterized by impaired glucose metabolism, insulin resistance, and progressive insulin deficiency. Changes in glucose homeostasis in T2DM create a continuous state of hyperglycemia in blood plasma. If glycemia is not controlled through the course of the disease, chronic hyperglycemia may lead to various long-term complications (macrovascular and microvascular), which lead to poor quality of life, increased morbidity, and mortality [2,3].

Based on the clinical guidelines from the American Diabetes Association and European Association for the Study of Diabetes, glycemic control of T2DM is done by combining lifestyle changes and pharmacological intervention through a stepwise approach. The first line of pharmacological treatment for T2DM is oral hyperglycemic agents. However, in cases of progressive beta-cell destruction and insulin deficiency where glycemic control could not be adequately achieved by only using oral anti glycemic agents, treatment is intensified by adding basal insulin therapy or glucagon-like peptide-1 (GLP-1) receptor agonist. One of the challenges of basal insulin therapy is the control of postprandial glucose levels. When postprandial glucose levels still spike even after constant basal insulin therapy, post-prandial or bolus insulin injection may be added to treatment regimens [2,3].

The use of basal and postprandial insulin may be effective in controlling blood glucose levels; however, it requires more daily injections and increases the cost of treatment, risk of hypoglycemia, and risk of error in usage by patients. These drawbacks may be inconvenient for most patients, as injections are uncomfortable for most. Aside from the inconveniences, an increased number of injections leads to reduced treatment compliance, thus hampering the effectiveness of glycemic control. Patients may come to the hospital even more morbidly than before their treatment was intensified, only because they missed most of their insulin injections [2,3].

As a solution to this, several pre-mixed or self-mixed insulin solutions were innovated. Although most of the time coformulation of basal and bolus insulin is impossible due to substance incompatibility, some long-acting and rapid-acting insulin combinations have been formulated. One of them is insulin degludec/insulin aspart (IDegAsp), a soluble co-formulation consisting of 70% basal insulin degludec and 30% postprandial insulin aspart [2,3].

IDegAsp is a potential T2DM treatment due to its glycemic coverage and reduced number of injections. However, to date, there has not been any comparison of IDegAsp with standard treatments (basal insulin or basal and bolus insulin) stratified by its frequency. A review of IDegAsp’s efficacy is needed to give a clear risk and benefit consideration for clinical practitioners in prescribing insulin therapies. Therefore, we created a systematic review comparing the efficacy and safety of IDegAsp to that of basal insulin regimen and basal plus bolus insulin regimen, stratified by its frequency, once daily or twice daily.

## Review

Materials and methods

**Figure 1:**
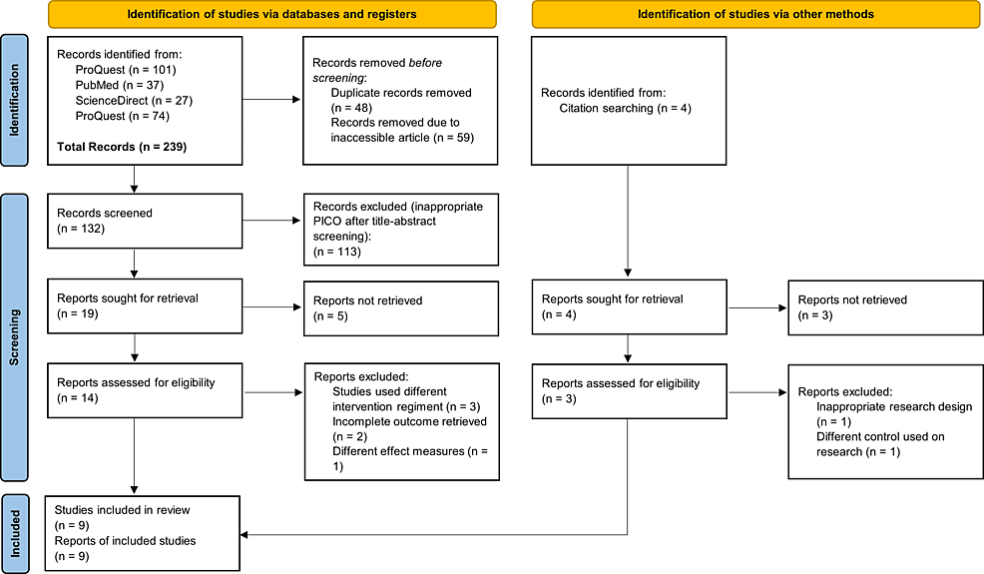
PRISMA statement flowchart on the literature search and selection process PRISMA: Preferred Reporting Items for Systematic Reviews and Meta-Analyses

We conducted a systematic review complying with the Preferred Reporting Items for Systematic Reviews and Meta-Analyses (PRISMA) guidelines to determine the efficacy and safety of IDegAsp in the management of T2DM [4]. The literature search and screening method were then summarized in the PRISMA statement flowchart in Figure 1.

Search Strategy

The search strategy was conducted independently by three reviewers (BCE, LW, and JRT) to ensure the reviewers’ objectivity. The search was conducted through PubMed, ProQuest, Scopus, and ScienceDirect on October 29, 2020. Each difference in search results was discussed further between the reviewers to make a decision. We also conducted hand searching with citation searching from the previously included studies to look for literature on IDegAsp and Type 2 DM. The search was conducted using search queries and keywords of (“Type 2 Diabetes Mellitus” OR “Type 2 DM”) AND “IDegAsp” in every database. A Medical Subject Headings (MeSH) term was used if it is available in the selected databases. Moreover, we contacted experts and researchers from the field to look for potential unscreened data and searched clinical trials on clinicaltrial.gov to look for the latest publication on IDegAsp and T2DM.

Study Selection

We selected and screened the studies using inclusion and exclusion criteria that preceded the research. All screening processes were done using Google Spreadsheet (Google LLC, Mountain View, California, United States). The study selection was done based on the predetermined patient, intervention, control, and outcome (PICO) criteria, in which we used T2DM as the targeted patient, IDegAsp as intervention, other insulin therapy as the control (insulin glargine, insulin aspart, etc.), and primary outcomes of insulin use, that is the changes in hemoglobin A1C (HbA1C) value (for efficacy) and hypoglycemia incident rate (for safety). We included studies with several inclusion criteria, including (1) randomized clinical trials, (2) studying a population of T2DM at any age, (3) using IDegAsp as an intervention or independent variable, (4) using another insulin as a controlled variable, (5) analyzing the outcome of IDegAsp use in T2DM, such as HbA1C changes and hypoglycemia events that compared with the control insulin, and (6) written in English. We also did citation alerts from existing systematic reviews or meta-analyses to increase the sensitivity of the acquired studies. However, we excluded editorial/review articles and case reports/case series as they were not suitable for this review. Articles on animal studies (non-human studies), inaccessible full-text articles, and non-English articles were also excluded. The selection process was conducted by adhering to PRISMA guidelines, starting from the title and abstract screening, followed by full-text screening. All screening processes were done independently by all investigators (BCE, LW, JRT). Every discrepancy or uncertainty was discussed by all investigators.

Quality Assessment

All acquired studies were then assessed for their quality using tools for critical appraisal by the Centre for Evidence-Based Medicine (CEBM) of the University of Oxford [5]. The critical appraisals were done in three different sections: validity, importance, and applicability. We also conducted a risk-of-bias assessment using Cochrane’s tool for risk-of-bias on seven different aspects of bias, which included selection bias (random sequence generation and allocation concealment), performance bias (blinding of participants and personnel), attrition bias (incomplete outcome data), detection bias (blinding of outcome assessment), and reporting bias (selective reporting) [6]. The critical appraisal and risk-of-bias assessment were conducted by three independent reviewers, with any disparities and differences discussed properly to make a final decision, and extrapolated into tables.

Data Extraction

Out of the selected studies, we extracted the information of study author, year, design, location, age of samples, sample size, intervention, control, the aim of the study, the primary endpoint, level of evidence (based on CEBM level of evidence 2011 [7]), and the length of each included study (the time period of data retrieval). The selected outcomes recorded in the review comprised HbA1C changes, proportions of participants achieving normal HbA1C (7%), hypoglycemia incident rate, nocturnal hypoglycemia incident rate, weight gain, and fasting glucose changes. The data were then extrapolated into tables and forest plots.

Meta-Analysis

All included studies were then included for quantitative analysis. The meta-analysis was done using Review Manager (RevMan) Version 5.4 (2020; The Cochrane Collaboration, London, England). All recorded outcomes were used in the pooled analysis, consisting of HbA1C changes, weight gain, and fasting glucose changes, which were reported in estimated treatment differences (ETD) value; proportions of participants achieving normal HbA1C, which were reported in odds ratio (OR); hypoglycemia incident rate and nocturnal hypoglycemia incident rate, which were reported in risk ratio (RR). Summary data and related 95% confidence interval (CI) were then calculated by conventional meta-analysis. Quantitative analysis for HbA1C changes, weight gain, and fasting glucose was conducted using fixed-effect inverse variance with continuous type of data with effect measure of mean difference. Analysis of proportions of participants achieving HbA1C changes was done using the fixed-effect Mantel-Haenszel method with dichotomous type of data with effect measure of OR. Meanwhile, analysis of hypoglycemia and nocturnal hypoglycemia incident rate was conducted using fixed-effect inverse variance with generic inverse variance type of data with effect measures of RR. All the analyses were conducted by dividing the included studies into four different subgroups, comprising “IDegAsp vs Once-daily Insulin Glargine (IGlar OD)” group, “IDegAsp vs Once-daily Insulin Glargine and Insulin Aspart (IGlar+IAsp OD)" group, “IDegAsp vs Bi-daily Insulin Aspart (BiAsp BID)” group, and “IDegAsp vs Bi-daily Insulin Degludec and Insulin Aspart (IDeg+IAsp BID)” group based on the frequency and control regiment of each study. All results were then visualized into forest plots and funnel plots. The indexes of heterogeneity (X2 or Q according to Cochran, I^2^, and tau^2^) were also calculated to analyze data distribution in each study. All analyses were made using the predetermined p-value of below 0.05 to be considered as significant.

Results

Study Selection and Study Characteristics

According to the search strategy, we found 10 studies eligible for analysis out of 132 articles initially meeting the search criteria from various databases (Figure 1). There are three studies comparing once-daily administration of IDegAsp to once-daily administration of insulin glargine (IGlar) [3,8,9], one study comparing once-daily administration of IDegAsp to once-daily administration of combined insulin glargine and insulin aspart (IAsp) [2], five studies comparing twice a day administration of IDegAsp to twice a day administration of biphasic insulin aspart (BIAsp) [10-14], and one study comparing twice a day administration of IDegAsp to twice a day administration of IDeg and IAsp [15]. All studies were randomized controlled studies, with some done in phase III [9,11-13,15]. There were seven multinational and intercontinental studies [2,3,8,10-12,15]. All studies involved patients aged 18 years old and above with a sample size of more than 100 for each study. Table 1 gives the details of the selected studies with complete characteristics.

**Table 1:** Characteristics of included studies IDegAsp: insulin degludec/insulin aspart; IGlar: insulin glargine; IAsp: insulin aspart; BiAsp: bi-daily insulin apart; OD: once-daily; BID: *bis in die* (twice a day); RCT: randomized controlled trial; FPG: fasting plasma glucose; SMPG: self-measured plasma glucose; ETD: estimated treatment difference; HbA1c: hemoglobin A1C

Author	Year	Design	Location	Age (years)	Sample size	Objective	Primary Endpoint	Level of Evidence	Length (weeks)
IDegAsp OD compared to IGlar OD									
Kumar et al. [3]	2016	RCT	Croatia, France, India, Poland, South Africa, South Korea, Sweden, Turkey, United States	>18	465	HbA1c mean change, FPG change, SMPG 9 point, overall prandial glucose increment	HbA1c mean change	1b	26
Kumar et al [8]	2016	RCT	Austria, India, Poland, Russia, South Korea, Spain, Turkey, United States	>18	413	HbA1c change, FPG change, PPG increment SMPG, number of participants with normal HbA1c, hypoglycemic episodes	HbA1c change	1b	26 + 26
Onishi et al. [9]	2013	RCT Phase III	Japan	>20	296	HbA1c change, FPG change, SMPG nine-point, total daily insulin dose, hypoglycaemic episodes, body weight	HbA1c change	1b	26
IDegAsp OD compared to IGlar + IAsp OD									
Tsimikas et al. [2]	2019	RCT	Algeria, Czech, India, Russia, Serbia, Turkey, US	>18	532	HbA1c mean change, FPG change, proportion of normal HbA1c, SMPG profile, total daily insulin dose	HbA1c change	1b	26
IDegAsp BID compared to BIAsp BID									
Kaneko et al. [10]	2015	RCT	Hong Kong, Japan, Malaysia, South Korea, Taiwan	>18	424	HbA1c change, FPG change, 9 point SMPG, body weight, proportion achieving normal HbA1c	HbA1c change	1b	26
Fulcher et al. [11]	2014	RCT Phase IIIa	Australia, Denmark, Finland, India, Malaysia, Poland, Sweden, Taiwan, Thailand, Turkey	>18	447	HbA1c change, FPG change, SMPG profile, proportion achieving normal hbA1c, hypoglycemic episodes, body weight, insulin dose	HbA1c change	1b	26
Taneda et al. [12]	2016	RCT Phase III	Hong Kong, Japan, Malaysia, South Korea, Taiwan	-	178	HbA1c change, FPG change, SMPG profile, proportion achieving normal hbA1c, hypoglycemic episodes, body weight, insulin dose	HbA1c change	1b	26
Yang et al. [13]	2019	RCT Phase III	China	>18	543	HbA1c change, FPG change after 26 w, nocturnal hypoglycemia, body weight change, response without hypoglycaemic episodes	HbA1c change	1b	26
Franek et al. [14]	2016	RCT	Europe	>18	371	Safety & efficacy of IDegAsp	Mean HbA1C, Events rate of hypoglycemia (nocturnal & general); ETD; Fasting glucose plasma	1b	26
IdegAsp BID compared to IDeg + IAsp BID									
Rodbard et al. [15]	2015	RCT Phase III	Algeria, Austria, France, Norway, United States	>18	274	HbA1c mean change, FPG change, proportion of normal HbA1c, SMPG profile, total daily insulin dose	HbA1c mean change	1b	26

The primary outcome of the included studies includes HbA1C mean changes, fasting plasma glucose (FPG) changes, proportions of participants achieving normal HbA1C, nine-point self-measured plasma glucose (SMPG), hypoglycemic episodes, daily insulin dose, and body weight increment. Most studies were conducted for a span of 26 weeks, except for the study by Kumar et al., which was for 52 weeks [8]. However, the outcome extracted from the study still includes the 26-weeks result of the study to ensure similar characteristics for all samples.

Quality Assessment

We found out that eight studies were excellent in terms of validity and applicability according to critical appraisal (Table 2), except for one study [12], which did not state equal treatment of samples in the trial, and thus concluded as unclear.

**Table 2:** Critical appraisal results NI: no information available on the included study

	Validity					Importance	Applicability	
Study	Randomized assignment of patient	Similar characteristics of samples	Equal treatment of samples	Minimal loss-to-follow up and intention-to-treat analysis	Double-blind analysis	Treatment effect & precision	Internal validity (PICO)	Patient similarity
Kumar et al. [3]	Yes	Yes	Yes	Yes	Unclear	NI	Yes	Yes
Kumar et al. [8]	Yes	Yes	Yes	Yes	Unclear	NI	Yes	Yes
Onishi et al. [9]	Yes	Yes	Yes	Yes	Unclear	NI	Yes	Yes
Tsikimas et al. [2]	Yes	Yes	Yes	Yes	Unclear	NI	Yes	Yes
Yang et al. [13]	Yes	Yes	Yes	Yes	Unclear	NI	Yes	Yes
Franek et al. [14]	Yes	Yes	Yes	Yes	Unclear	NI	Yes	Yes
Rodbard et al. [15]	Yes	Yes	Yes	Yes	Unclear	NI	Yes	Yes
Kaneko et al. [10]	Yes	Yes	Yes	Yes	Unclear	NI	Yes	Yes
Fulcher et al. [11]	Yes	Yes	Yes	Yes	Unclear	NI	Yes	Yes
Taneda et al. [12]	Yes	Yes	Unclear	Yes	Unclear	NI	Yes	Yes

Based on the Cochrane risk-of-bias assessment, all studies were clear of selection bias, attrition bias, reporting bias, and other biases (Table 3). However, blinding of all studies was unclear because these studies have objective measures, thus blinding was not necessary.

**Table 3:** Risk-of-bias assessment results L*: *no risk-of-bias found; U: unclear risk-of-bias

Study	Random sequence generation (selection bias)	Allocation concealment (selection bias)	Blinding of participants and personnel (performance bias)	Blinding of outcome assessment (detection bias)	Incomplete outcome data (attrition bias)	Selective reporting (reporting bias)	Other bias
Kumar et al. [3]	L	L	U	U	L	L	L
Kumar et al. [8]	L	L	U	U	L	L	L
Onishi et al. [9]	L	L	U	U	L	L	L
Tsimikas et al. [2]	L	L	U	U	L	L	L
Yang et al. [13]	L	L	U	U	L	L	L
Franek et al. [14]	L	L	U	U	L	L	L
Rodbard et al. [15]	L	L	U	U	L	L	L
Kaneko et al. [10]	L	L	U	U	L	L	L
Fulcher et al. [11]	L	L	U	U	L	L	L
Taneda et al. [12]	L	L	U	U	L	L	L

Efficacy of IDegAsp: Glycemic Index

We found that IDegAsp administration once a day gave better HbA1c change with an estimated treatment difference (ETD) of -0.28% (95% CI -0.46; -0.10) as compared to IGlar, according to one study as seen in Table 4 [9].

**Table 4:** Outcome of included studies IDegAsp: insulin degludec/insulin aspart; IGlar: insulin glargine; IAsp: insulin aspart; BiAsp: bi-daily insulin aspart; OD: once-daily; BID: *bis in die* (twice a day); ETD: estimated treatment difference; OR: odds ratio; RR: risk ratio; HbA1c: hemoglobin A1C

Author	HbA1c Change	Proportion of participants achieving normal HbA1c (<7%)	Hypoglycemia incident	Nocturnal hypoglycemia incident	Weight Gain	Fasting Glucose	Other
IDegAsp OD compared to IGlar OD							
Kumar et al. [3]	ETD -0.03% (95% CI -0.20; 0.14)	OR 1.18 (95% CI 0.78; 1.78)	RR 1.43 (95% CI 1.07; 1.92)	RR 0.80 (95% CI 0.49; 1.30)	ETD 0.33 kg (95% CI -0.17; 0.83)	ETD 0.33 mmol/l (95% CI -0.11; 0.77)	
Kumar et al. (week 26, 52) [8]	ETD -0.08% (95% CI -0.26; 0.09)	OR 0.95 (95% CI 0.66; 1.35)	TR 1.96 (95% CI 1.42; 2.44)	TR 0.25 (95% CI 0.15; 0.407)	ETD 1.60 kg(95% CI 0.84; 2.36)	ETD 0.28 mmol/l (95% CI -0.14; 0.69)	
Onishi et al. [9]	ETD -0.28% (95% CI -0.46; -0.10)	OR 2.21 (95% CI 1.25; 3.92)	RR 0.73 (95% CI 0.50; 1.08)	RR 0.75 (95% CI 0.34; 1.64)	N/A	ETD -0.15 mmol/l (95% CI -0.29; 0.60)	
IDegAsp OD compared to IGlar + IAsp OD							
Tsimikas et al. (week 26 and 38) [2]	ETD 0.07% (95% CI -0.06; 0.21)	OR 1.07 (95% CI 0.74; 1.54)	RR 0.90 (95% CI 0.67; 1.22)	RR 0.55 (95% CI 0.34; 0.90)	ETD 0.43 (95% CI -0.13; 0.99)	ETD 0.04 (-0.34; 0.42)	
IDegAsp BID compared to BIAsp BID							
Kaneko et al. [10]	ETD 0.05% (95% CI -0.10; 0.20)	OR 0.94 (95% CI 0.61; 1.44)	RR 1.00 (95% CI 0.76; 1.32)	RR 0.67 (95% CI 0.43; 1.06)	ETD -0.38 kg (95% CI -0.96; 0.21)	ETD -1.06 mmol/l (95% CI -1.43; -0.70)	
Fulcher et al. [11]	ETD -0.03% (95% CI -0.18; 0.13)	N/A	RR 0.68 (95% CI 0.52; 0.89)	RR 0.27 (95% CI 0.19; 0.41)	ETD -0.62 kg (95% CI -1.15; -0.10)	ETD -1.14 mmol/l (95% CI -1.53; -0.76)	Lower insulin dose (RR = 0.89; 95% CI 0.83; 0.96)
Taneda et al. [12]	ETD -0.13% (95% CI -0.31; 0.04)	OR 1.20 (95% CI 0.59; 2.46)	RR 1.63 (95% CI 0.66; 4.06)	RR 0.44 (95% CI 0.20; 0.99)	ETD -0.14 kg (95% CI -1.01; 0.74)	ETD -1.50 mmol/l (95% CI -1.98; -1.01)	
Yang et al. [13]	ETD -0.08% (95% CI -0.20; 0.05)	OR 2.22 (95% CI 1.47; 3.35)	RR 0.57 (95% CI 0.42; 0.77)	RR 0.53 (95% CI 0.33; 0.87)	ETD 0.61 kg (95% CI 0.15; 1.08)	ETD -1.42 mmol/l (95% CI -1.74; -1.10)	Lower insulin dose of 20%
Franek et al. [14]	ETD 0.02% (95% CI -0.12; 0;17)	N/A	RR 0.46 (95% CI 0.35; 0.61)	RR 0.25 (95% CI 0.16; 0.38)	ETD 0.79 kg (95% CI -0.03; 1.61)	ETD -1.00 mmol/l (95% CI -1.40; -0.60)	
IdegAsp BID compared to IDeg + IAsp BID							
Rodbard et al. [15]	ETD 0.18% (95% CI -0.04; 0.41)	OR 0.50 (95% CI 0.50; 1.38)	RR 0,82 (95% CI 0.61; 1.07)	RR 0.80 (95% CI 0.50; 1.29)	ETD -1.04 kg (95% CI -1.99; -0.10)	ETD -0.31 mmol/l (95% CI -0.97; 0.34)	Lower total daily insulin dose (107 U vs 131U) after 26 weeks

Two studies have shown more patients achieving <7% HbA1c in 26 weeks in the once-daily IDegAsp group, with OR of 1.18 (95% CI 0.78; 1.78) [8] and 2.21 (95% CI 1.25; 3.92) [9]. Once-daily administration of IDegAsp compared to once-daily IGlar + IAsp showed no significant reduction of HbA1c levels, weight gain, and fasting glucose levels. However, the number of participants reaching normal levels of HbA1c in IDegAsp compared to IGlar + IAsp is significant with an OR of 1.07 (95% CI 0.74; 1.54). Twice a day IDegAsp administration showed no inferiority, nor superiority compared to BIAsp in terms of HbA1c reduction [10-14]. More patients achieved normal HbA1c levels with bi-daily IDegAsp in three out of four studies assessing this outcome, with OR 1.60 (95% CI 0.94; 2.72) [11], OR 1.20 (95% CI 0.59; 2.46) [12], and OR 2.22 (95% CI 1.47; 3.35) [13]. Moreover, there were three studies [11,13,14] that stated a lower risk of hypoglycemia incident in twice a day IDegAsp administration with the lowest RR of 0.46 (95% CI 0.35; 0.61) fold. In addition, five studies showed that twice a day IDegAsp administration showed more reduction in fasting glucose compared to BIAsp [10-14], with the largest margin ETD of -1.50 (95% CI -1.98; -1.01) mmol/l. Two studies reported there was a lower insulin dose of twice-daily IDegAsp up to 20% compared to BIAsp by week 26 of administration [11,13].

**Figure 2:**
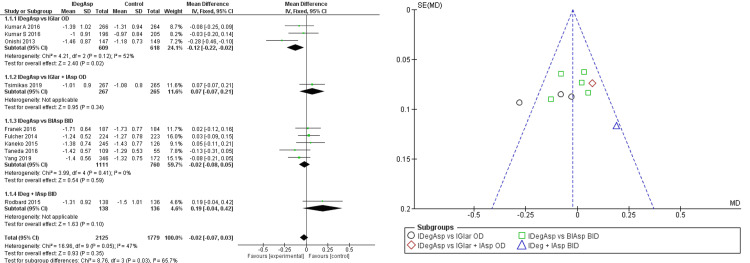
Forest plot and funnel plot of pooled analysis on HbA1C changes IDegAsp: insulin degludec/insulin aspart; IGlar: insulin glargine; IAsp: insulin aspart; BiAsp: bi-daily insulin aspart; OD: once-daily; BID: *bis in die* (twice a day) The forest plot uses effect measure of mean estimated treatment difference (ETD) [2,3,8-15]

Based on our meta-analysis, we found a significant HbA1c reduction in administration of IDegAsp compared to IGlar once daily up to -0.12 (95% CI -0.22, -0.02) (Figure 2).

**Figure 3:**
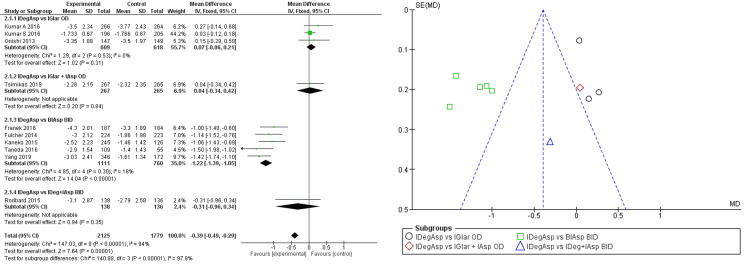
Forest plot and funnel plot of pooled analysis on FPG changes IDegAsp: insulin degludec/insulin aspart; IGlar: insulin glargine; IAsp: insulin aspart; BiAsp: bi-daily insulin aspart; OD: once-daily; BID: *bis in die* (twice a day); FPG: fasting glucose plasma The forest plot uses effect measure of mean estimated treatment difference (ETD) [2,3,8-15]

In addition, better fasting glucose changes were seen significantly in IDegAsp with a mean difference of -0.31 (95% CI -0.49; -0.29) (Figure 3).

**Figure 4:**
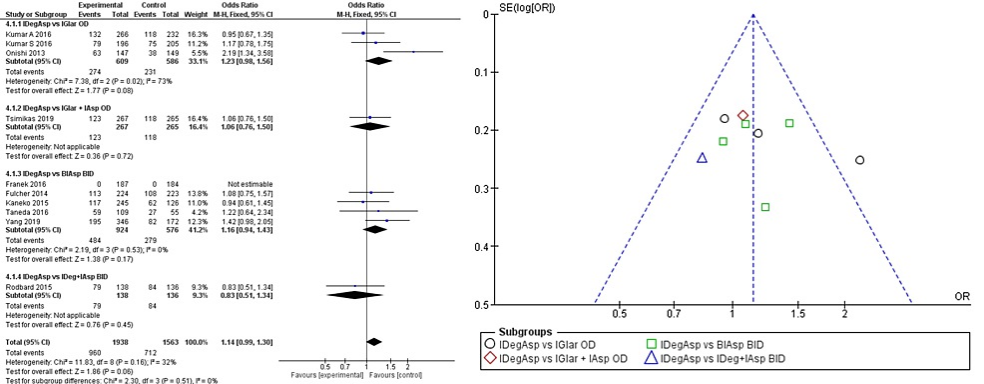
Forest plot and funnel plot of pooled analysis on proportions of participants achieving normal HbA1C IDegAsp: insulin degludec/insulin aspart; IGlar: insulin glargine; IAsp: insulin aspart; BiAsp: bi-daily insulin aspart; OD: once-daily; BID: bis in die (twice a day) The forest plot uses effect measure of mean estimated treatment difference (ETD) [2,3,8-15]

It was also seen when compared to BIAsp twice daily with a mean difference of -1.22 (95% CI -1.39; -1.05). However, the significance of patients reaching normal HbA1c levels in the IDegAsp group still could not be determined (OR 1.14; 95% CI 0.99; 1.30) under homogeneously distributed data, while stating the superiority of IDegAsp in the analysis (Figure 4).

Safety of IDegAsp: Hypoglycemia incident and weight gain increment

**Figure 5:**
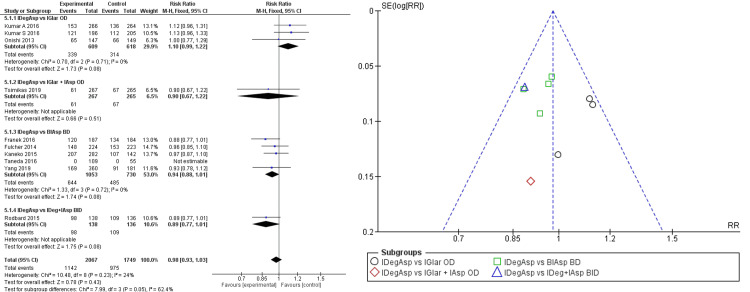
Forest plot and funnel plot of pooled analysis on overall hypoglycemia incident rate IDegAsp: insulin degludec/insulin aspart; IGlar: insulin glargine; IAsp: insulin aspart; BiAsp: bi-daily insulin aspart; OD: once-daily; BID: *bis in die* (twice a day) The forest plot uses effect measure of mean estimated treatment difference (ETD) [2,3,8-15]

In terms of safety, once-daily administration of IDegAsp resulted in lower rate of incidence of overall hypoglycemia compared to once-daily IGlar + IAsp (RR 0.90 (95% CI 0.67; 1.22)), bi-daily IAsp (RR 0.94 95% (CI 0.88; 10.01)), and bi-daily IAsp + IDeg (RR 0.89 (95% CI 0.77; 1,01)). In contrast, compared to once-daily IGlar, IDegAsp showed a higher incidence of overall hypoglycemia (RR 1.10 (95% CI 0.99; 1.22)) (Figure 5). Pooled analysis of every subgroup resulted in a better IDegAsp safety profile in overall hypoglycemia (RR 0.98 (95% CI 0.93; 1.03)). However, pooled analysis and subgroup analysis of overall hypoglycemia showed wide confidence intervals, under homogeneously distributed data. All outcomes also showed a nonsignificant difference between the two comparisons, hence it might only be stated as indicative.

**Figure 6:**
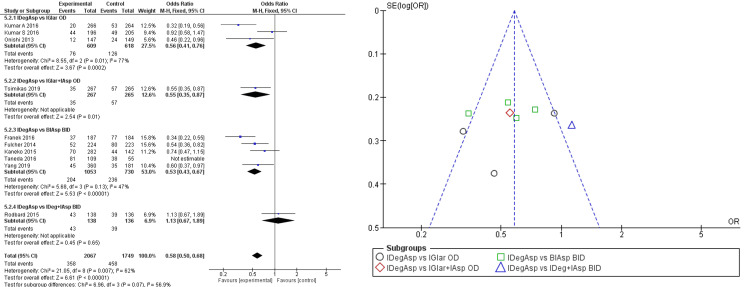
Forest plot and funnel plot of pooled analysis on nocturnal hypoglycemia incident rate IDegAsp: insulin degludec/insulin aspart; IGlar: insulin glargine; IAsp: insulin aspart; BiAsp: bi-daily insulin aspart; OD: once-daily; BID: *bis in die* (twice a day) The forest plot uses effect measure of mean estimated treatment difference (ETD) [2,3,8-15]

Subgroup analysis showed statistically significant lower risk of nocturnal hypoglycemia in IDegAsp administration compared to once-daily IGlar (RR 0.56 (95% CI 0.41; 0.76)), once-daily IAsp (RR 0.55 (95% CI 0.35; 0.87)), and bi-daily IAsp (RR 0.53 (95% CI 0.43; 0.67)) (Figure 6).

There were also four studies that reported a lower incidence of nocturnal hypoglycemia in IDegAsp administration twice daily with the lowest RR of 0.25 (95% CI 0.16; 0.38) fold [11-14]. According to one study, we also found a lower incidence of nocturnal hypoglycemia in the once-a-day IDegAsp group compared to the IGlar group by 75% [8]. Comparison with bi-daily IDeg+IAsp (different formulation) consisting of one study showed inferior results of IDegAsp administration to nocturnal hypoglycemia (RR 1.13 (95% CI 0.67; 1.89)), though statistically insignificant. Pooled analysis of subgroups showed a statistically significant reduction of nocturnal hypoglycemia in the administration of IDegAsp (RR 0.58 (95% CI 0.50; 0.68)).

**Figure 7:**
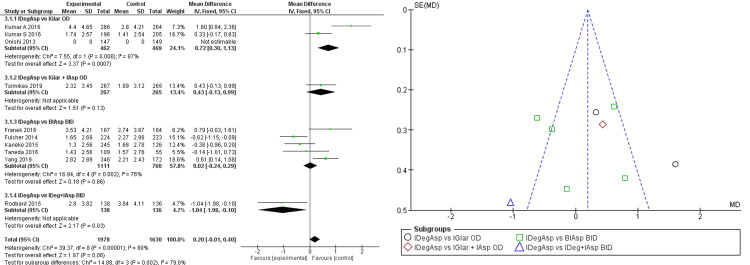
Forest plot and funnel plot of pooled analysis on weight gain increment IDegAsp: insulin degludec/insulin aspart; IGlar: insulin glargine; IAsp: insulin aspart; BiAsp: bi-daily insulin aspart; OD: once-daily; BID: *bis in die* (twice a day) The forest plot uses effect measure of mean estimated treatment difference (ETD) [2,3,8-15]

The result of weight gain varied between subgroups as shown in Figure 7.

Administration of once-daily IDegAsp showed higher weight gain compared to once-daily IGlar (ETD 0.72 (95% CI 0.43; 0.67)) with non-homogenous data distribution, to once daily IAsp (ETD 0.43 (95% CI -0.13; 0.99)). Bi-daily administration of IDegAsp also showed higher weight gain compared to bi-daily IAsp (ETD 0.22 (95% CI -0.24; 0.29)). However, administration of IDegAsp co-formulation in one study could decrease weight gain incidence compared to its separate formulation (ETD -1.04 (95% CI -1.98; -0.10)). Pooled analysis showed a higher incidence of weight gain in IDegAsp administration; however, the data is deemed inconclusive due to wide confidence intervals, the variance between subgroups, and high heterogeneity. 

Discussion

IDegAsp is the first soluble co-formulation insulin and comprises two insulin analogues that cover both basal and prandial glycemic control [16,17]. As a co-formulation of both basal and prandial insulin, IDegAsp has become the solution to the quandary of first-time insulin users who had to choose between basal and prandial. The insulin itself has also reported quite a positive outcome.

Once-daily IDegAsp administration gave a better HbA1c reduction compared to once-daily IGlar, which could not be explained clearly by twice-daily IDegAsp administration [9-14]. It was also determined that once-daily IDegAsp compared to once-daily IGlar was the only subgroup with a significant difference in our meta-analysis (mean difference -0.12; 95% CI -0.22; -0.02). It was similar to one study that there was HbA1c elevation in the first three months of once-daily basal insulin administration (8.70%±1.00%; p<0.05) and HbA1c reduction in the first three months of changing into once-daily IDegAsp (8.28%±1.10%; p<0.05) with significant effect up to six months of the administration [18]. There was a study that stated that IDegAsp administration was not linked with the difference in HbA1c in insulin-naive patients; however, the same study stated there was a reduction of FPG by 1.0 mmol/l (p<0.05), which could not be determined in all studies included [8]. This mechanism could be explained by the fact that IDegAsp consists of IDeg di-hexamers and IAsp hexamers. IDeg di-hexamers create a pool of soluble multi-hexamers that continuously form into monomers, thus slowly dissociating. Meanwhile, IAsp hexamers dissociate swiftly to monomers. IDeg has a half-life of 25.3 hours with a duration of action of up to 42 hours, while IAsp acts rapidly in 10-15 minutes of onset and reaches peak action in 90 minutes [8,19]. Due to its sustainability and bioavailability, once-daily IDegAsp was proven enough to lower HbA1c levels. These traits were also proven on lowering fasting glucose levels, which was proven further by our meta-analysis (mean difference -0.31; 95% CI -0.49; -0.29). Therefore, it is currently known that IDegAsp is considered superior to certain other insulin in a matter of both short-term and long-term actions in lowering blood glucose levels and HbA1c.

Once-daily IDegAsp was neither superior nor inferior to twice daily IDegAsp in the number of patients achieving normal HbA1c levels compared to only basal or only bolus insulin, as the majority of studies showed more proportion of patients reaching physiologic HbA1c on 26-week IDegAsp therapy. In addition, our meta-analysis has shown no signs of IDegAsp working better than other insulin in normalizing HbA1c (OR 1.14; 95% CI 0.99; 1.30). However, co-formulation of bi-daily IDegAsp is not superior to separate bi-daily IDeg + IAsp in the proportion of patients achieving the HbA1c target [15]. This may be due to the flexibility of dosing and titration in separate IDeg + IAsp. In premixed IDegAsp, the proportion of IDeg and IAsp is always fixed to 30:70, leaving less room for individualized treatment [10]. Meanwhile, separate regiments, though inconvenient in nature, are more personalized to the patients’ individual fluctuations of fasting and point glucose levels [10]. The treatment period also affected the course of treatment, as similar studies eventually reach superior results in 52 weeks of treatment [10]. 

In the terms of safety, we have found that IDegAsp's long-acting trait did not improve the risk of nocturnal hypoglycemia as we saw that there is a reduction of nocturnal hypoglycemia in one study [8]; with all studies showing statistically insignificant results in the superiority of IDegAsp. Nocturnal hypoglycemia incidence reduction was achieved in both once-daily and twice-daily administration of IDegAsp in comparison to IAsp and IGlar; although a separate formulation of IDeg+IAsp showed better results compared to the IDegAsp co-formulation [8,11-14]. This was contributed by the stability of IDeg di-hexamers so that impact of rapid and slow-acting insulin was clearly separated. It is known by the same study that IDegAsp metabolism and excretion were not impaired by renal or liver impairment at any level [20]. Therefore, there was a lower risk of IDegAsp-related toxicity. However, there is a higher risk of hypoglycemia in once-daily administration of IDegAsp according to two studies [3,8]. It was stated that hypoglycemic events peaked in the evening, between 20:00 and 24:00 with the majority of subjects taking a once-daily IDegAsp post the evening meal. In contrast, once-daily IGlar administration post evening meals showed hypoglycemic effects between 04:00 and 08:00 [3]. These phenomena were linked with the onset activity of IDegAsp and IGlar, which differed. IDegAsp showed a peak glucose-lowering effect four hours after administration and slowed up to 24 hours after administration [21]. Therefore, to achieve a reduction of HbA1c using once-daily IDegAsp without risking hypoglycemia, it is recommended to give an initial dose at the largest mealtime with tapering if needed. It has been proven that hypoglycemia incidence was significantly reduced by IDegAsp administration on the largest meal of the day. It also gave better FPG reduction after 26 weeks [8].

Hypoglycemia also could be reduced by using a stepwise titration scheme [22]. As an alternative, three studies suggested that twice a day IDegAsp administration could lower the risk of hypoglycemia based on a lower dose [11,13,14], which could be reduced to 80% of the basal insulin dose according to two studies [13,14]. Twice a day IDegAsp administration also showed a greater effect in FPG reduction in once-daily administration, according to five studies [10-14]. However, there was a study stating the inconvenience of twice daily insulin contributed to 6% of non-adherence to insulin administration protocol [23]. Therefore, twice-daily administration of IDegAsp should be re-evaluated even though there is a lower risk of hypoglycemia compared to once-daily administration.

The relation between IDegAsp administration, both once daily and twice daily, and weight gain could not be determined due to its wide CI and high heterogeneity [8,11,13]. This finding correlated with two other studies [9,14], which found weight gain in both arms of IDegAsp and basal insulin. One study mentioned before stated weight gain as an adverse effect of IDegAsp administration with a high dose on patients with a higher risk of hypoglycemia [8]. Therefore, there was a relation between dosing and weight gain in IDegAsp administration, both once daily and twice daily, with other types of insulin showing similar weight gain effects.

This is a systematic review that positioned various frequencies of IDegAsp administration among other established insulin administration in terms of efficacy and safety. However, this study involved some phase III randomized controlled trials (RCTs), considered by some as not eligible for quantitative review. Therefore, high-quality RCTs on the efficacy and safety of IDegAsp on certain populations should be done to provide more data for meta-analysis in order to produce pooled variables of IDegAsp’s efficacy and safety.

## Conclusions

IDegAsp once-daily administration was proven more beneficial in terms of HbA1c reduction and lowering nocturnal hypoglycemia incidence, but not hypoglycemia incidence and weight gain. On the other hand, IDegAsp twice-daily administration was not yet proven in HbA1c reduction and reduced weight gain but proven in lowering hypoglycemia incidence and nocturnal hypoglycemia incidence. Therefore, considering compliance and convenience, we suggest once-daily administration of IDegAsp with a stepwise titration scheme on the largest meal of the day to achieve benefit while minimizing risk. In addition, we would like to suggest more RCTs performed in order to provide more data for quantitative analysis of efficacy and safety.
